# A core role for cognitive processes in the acute onset and maintenance of post‐traumatic stress in children and adolescents

**DOI:** 10.1111/jcpp.13054

**Published:** 2019-03-25

**Authors:** Richard Meiser‐Stedman, Anna McKinnon, Clare Dixon, Adrian Boyle, Patrick Smith, Tim Dalgleish

**Affiliations:** ^1^ Department of Clinical Psychology University of East Anglia Norwich UK; ^2^ Centre for Emotional Health Department of Psychology Macquarie University Sydney NSW Australia; ^3^ Sussex Partnership National Health Service Foundation Trust Sussex UK; ^4^ Emergency Department Addenbrooke's Hospital Cambridge UK; ^5^ Department of Psychology Institute of Psychiatry, Psychology and Neuroscience King's College London London UK; ^6^ Medical Research Council Cognition and Brain Sciences Unit University of Cambridge Cambridge UK; ^7^ Cambridgeshire and Peterborough NHS Foundation Trust Cambridge UK

**Keywords:** Post‐traumatic stress disorder, cognitive development, longitudinal studies, early intervention

## Abstract

**Background:**

Post‐traumatic stress disorder (PTSD) is a common reaction to trauma in children and adolescents. While a significant minority of trauma‐exposed youth go on to have persistent PTSD, many youths who initially have a severe traumatic stress response undergo natural recovery. The present study investigated the role of cognitive processes in shaping the early reactions of child and adolescents to traumatic stressors, and the transition to persistent clinically significant post‐traumatic stress symptoms (PTSS).

**Methods:**

A prospective longitudinal study of youth aged 8–17 years who had attended a hospital emergency department following single trauma was undertaken, with assessments performed at 2–4 weeks (*N* = 226) and 2 months (*N* = 208) post‐trauma. Acute stress disorder and PTSD were assessed using a structured interview, while PTSS, depression severity and peritraumatic and post‐traumatic cognitive processes were assessed using self‐report questionnaires. On the basis of their PTSS scores at each assessment, participants were categorised as being on a resilient, recovery or persistent trajectory.

**Results:**

PTSS decreased between the two assessments. Cognitive processes at the 2‐ to 4‐week assessment accounted for the most variance in PTSS at both the initial and follow‐up assessment. The *onset* of post‐traumatic stress was associated particularly with peritraumatic subjective threat, data‐driven processing and pain. Its maintenance was associated with greater peritraumatic dissociation and panic, and post‐traumatic persistent dissociation, trauma memory quality, rumination and negative appraisals. Efforts to deliberately process the trauma were more common in youth who experienced the onset of clinically significant PTSS. Regression modelling indicated that the predictive effect of baseline negative appraisals remained when also accounting for baseline PTSS and depression.

**Conclusions:**

Cognitive processes play an important role in the onset and maintenance of PTSS in children and adolescents exposed to trauma. Trauma‐related appraisals play a particular role when considering whether youth make the transition from clinically significant acute PTSS to persistent PTSS.

## Abstract 2

Read the Commentary on this article at doi: 10.1111/jcpp.13081

## Introduction

Post‐traumatic stress disorder (PTSD) is a common response to trauma in children and adolescents (Alisic et al., 2014) that is prognostic of longer‐term deleterious impacts on mental health and functioning (Bolton et al., 2004; Morgan, Scourfield, Williams, Jasper, & Lewis, 2003). PTSD is a near‐unique disorder in that its onset can be linked to a particular event. However, mental health professionals and diagnostic systems typically steer away from diagnosing PTSD in the first month after a trauma as it is recognised that some degree of acute traumatic stress symptoms represents a normative response. Prospective studies of trauma‐exposed youth support this view, with considerable natural recovery occurring in the weeks and months following trauma (Hiller et al., 2016). The diagnosis of acute stress disorder (ASD) has been proposed by the American Psychiatric Association (APA) (2013) to identify individuals with high levels of clinically significant symptoms in these first 4 weeks post‐trauma, who are deemed at elevated risk of later PTSD.

Two important questions are then apparent: Why do only some young people have significant traumatic stress symptoms in the days and weeks immediately post‐trauma while others do not, and why do some youth then recover without treatment while others go on to suffer from persistent PTSD? Cognitive theorists propose that individual differences in the way the trauma is psychologically processed shape these differential responses and trajectories (Brewin, Dalgleish, & Joseph, 1996; Dalgleish, 2004; Ehlers & Clark, 2000). Central to these models is the contribution of peritraumatic cognitive processes that operate at the time of the trauma to the initial acute onset of traumatic stress symptoms. These processes include subjective experiences of threat and panic, ‘data‐driven processing’ (i.e. overwhelming sensory impressions and confusion during the trauma, where the individual has difficulty in making sense of the trauma as it occurs) and dissociation. Several additional post‐traumatic cognitive processes are then proposed to maintain post‐traumatic stress symptoms (PTSS) over time. These include the presence of a poorly elaborated, fragmented and sensory‐based memory of the trauma (as a function of the aforementioned peritraumatic processes), cognitive and behavioural avoidance of trauma‐related stimuli, negative appraisals of the self and world following the trauma (e.g. believing that one cannot cope, that one's reactions are a sign of permanent psychological damage or weakness), rumination (e.g. persistent thinking around difficult to resolve questions such as ‘why did this happen to me’, ‘what could I have done differently’) and persistent dissociation, which is proposed to impede the elaboration of trauma memories (Ehlers & Clark, 2000).

While some efforts have been made to consider the contribution of cognitive processes in youth, these studies have focused largely on the role of negative trauma‐related appraisals (Mitchell, Brennan, Curran, Hanna, & Dyer, 2017). While other cognitive mechanisms such as data‐driven processing (McKinnon, Nixon, & Brewer, 2008), trauma memory quality (Salmond et al., 2011) and rumination (Stallard & Smith, 2007) have also been shown to be associated with post‐traumatic stress, the studies were small and cross‐sectional in nature, and/or have used single‐item measures of the constructs of interest (Ehlers, Mayou, & Bryant, 2003; Stallard & Smith, 2007). The few studies that have utilised a prospective design have typically done so beyond the acute window (i.e. in the first days and weeks following a trauma) when most natural recovery would be anticipated to occur (Palosaari, Punamaki, Diab, & Qouta, 2013). The full range of cognitive processes proposed by cognitive models of PTSD has not been examined together within the same study in youth. Moreover, these cognitive processes have not been considered in the context of other plausible pre‐, peri‐ and post‐trauma predictors of onset and maintenance of traumatic stress symptoms, to clarify their independent role in driving psychiatric outcomes.

The current prospective longitudinal study of children and adolescents aged 8–17 years recently exposed to a traumatic stressor allowed for a comprehensive examination of this important issue. The study addressed symptoms of PTSD 2–4 weeks and 2 months following a trauma, when much natural recovery is still occurring. In particular, we sought to consider three questions. First, we examined whether cognitive processes would account for unique variance in PTSS over and above the effect of demographic factors, trauma nature and severity variables, and psychosocial factors not specifically implicated in cognitive models of PTSD (i.e. social support, life stressors). Second, we sought to investigate whether, consistent with cognitive models of PTSD, distinct cognitive mechanisms have a specific role in either the onset (e.g. peritraumatic factors such as subjective threat, panic and data‐driven processing) or maintenance (e.g. appraisals, rumination) of PTSS. To this end, we compared three groups: a ‘resilient’ group who did not develop clinically significant traumatic stress symptoms at either time point; a ‘recovery’ group who initially had clinically significant symptoms but not clinically significant symptoms at follow‐up; and a ‘persistent group’ who had clinically significant symptoms at both time points. Third, we also examined whether active attempts to process the trauma, for example talking the trauma through with friends or family, measured at the 2‐ to 4‐week assessment may be protective against PTSS at 2 months.

## Methods

### Participants

Participants were consecutive child and adolescent attendees (8–17 years) at four emergency departments (EDs) in the East of England following single‐event trauma between 3 September 2010 and 30 April 2013. An event was considered a ‘trauma’ if consistent with the DSM‐5 PTSD (APA, 2013) definition. An event was considered a ‘single event’ if it comprised a ‘one‐off’ incident unrelated to maltreatment. Exclusion criteria were inability to speak English, learning disability, attendance after deliberate self‐harm, being either under the care of social services or where a child protection issue was related to the presentation, and moderate‐to‐severe traumatic brain injury.

Of 773 eligible youth, 168 (21.7%) could not be contacted due to incomplete/inaccurate details. Of the 605 families who could be contacted, 315 (52.1%) declined to participate, 30 (5.0%) did not meet inclusion criteria, and 260 (43.0%) agreed to participate. Of these, 226 (37.4% of attendees contacted) completed the initial 2‐to 4‐week assessment (days since trauma, *M* = 22.0, *SD* = 7.2); the remainder only completed the 2‐month assessment and are not included here. Of the 226 participants who completed the initial assessment, only 8 (3.5%) did so more than 1‐month post‐trauma.

There were no significant differences between participants (*n* = 260) and eligible nonparticipants (*n* = 483, including children who could not be contacted) with respect to age, sex, ethnicity, number of injuries, being seen in ‘resus’, days admitted, previous attendances or head injury (*p*s > .05). Relative to nonparticipants, participants were more likely to report greater pain, be admitted to hospital, be admitted to paediatric intensive care, have lost consciousness and have been assaulted (all *p*s < .05).

Of the 226 participating youth at 2–4 weeks (mean 14.1 years, *SD* = 2.9), 96 (42.5%) were female and 16 (7.1%) belonged to a minority ethnic group. Trauma types included motor vehicle collision (*n* = 104; 46.0%); assault (*n* = 41; 18.1%); dog attack (*n* = 10; 4.4%); accidental injuries (*n* = 70; 31.0%); and medical emergency (*n* = 1; 0.4%). Further data on injury severity are displayed in Table 1.

**Table 1:** Correlations between predictor variables and post‐traumatic stress severity at each assessment

	Mean (*SD*)/Frequency (%)	PTSS (2–4 weeks)		PTSS (2 months)		Acute stress disorder diagnosis (2–4 weeks)		PTSD diagnosis (2 months)	
		*r*	*N*	*r*	*N*	*r*	*N*	*r*	*N*
Demographic variables									
Age	14.2 (2.9)	.12	197	.06	206	.08	208	.12	208
Female sex	89 (42.8%)	.13	197	.05	206	.04	208	−.02	208
Minority ethnicity	13 (6.3%)	−.07	197	.00	206	.07	208	−.02	208
Household income >£20K	127 (61.1%)	−.15*	172	−.05	179	−.05	180	−.16*	180
Pretrauma emotional well‐being									
Emotional difficulties	7.7 (3.1)	.43****	197	.35****	201	.29****	202	.25***	202
Trauma severity									
Assault (yes/no)	36 (17.3%)	.26***	197	.27****	206	.37****	208	.32****	208
Head injury	81 (38.9%)	.21**	195	.07	204	.19**	206	.19**	206
Number of injuries	1.7 (.9)	.07	197	.04	206	.03	208	.13	208
Fracture (yes/no)	47 (22.6%)	−.06	197	−.07	206	−.08	208	.02	208
Admission (yes/no)	60 (28.8%)	−.17	197	−.14	206	−.20	207	−.14	208
Resus (yes/no)	25 (12.0%)	−.09	195	−.06	204	−.11	206	−.07	206
Permanent loss function	7 (3.4%)	−.11	197	−.09	206	−.08	208	−.06	208
Week 2 psychopathology									
PTSS (CPSS)	11.5 (11.1)	‐	‐	.72****	196	.62****	197	.57****	197
Depression (SMFQ)	5.3 (5.8)	.78****	197	.63****	196	.50****	197	.53****	197
Two‐month psychopathology									
PTSS (CPSS)	7.7 (9.7)	‐	‐	‐	‐	‐	‐	.71****	206
Depression (SMFQ)	4.5 (5.5)	‐	‐	‐	‐	.68****	205	.53****	205
Peritrauma cognitive processing									
Threat	7.8 (2.6)	.51****	196	.36****	195	.28****	196	.21**	196
Data‐driven processing	15.8 (6.0)	.54****	196	.48****	195	.44****	196	.32****	196
Panic	3.7 (2.4)	.59****	196	.54****	195	.43****	196	.37****	196
Peritraumatic dissociation	4.0 (3.1)	.52****	196	.45****	195	.40****	196	.33****	196
Peritraumatic pain	3.1 (1.1)	.35****	195	.19**	194	.14*	195	.14*	195
Post‐trauma psychosocial factors (Week 2)									
Adaptive processing	13.6 (3.8)	.32****	196	.27***	196	.17*	196	.17*	196
Ongoing pain	1.7 (.9)	.38****	196	.32****	195	.28****	196	.18*	196
Life stressors	.9 (1.1)	.22**	196	.15*	205	.19**	207	.09	207
Social support (MSPSS)	69.4 (13.0)	−.09	197	−.07	195	−.02	197	−.03	197
Post‐trauma cognitive processing (Week 2)									
Persistent dissociation	1.5 (2.4)	.68****	197	.61****	196	.53****	197	.44****	197
Memory quality (TMQQ)	21.8 (6.9)	.70****	196	.62****	195	.47****	196	.47****	196
Negative appraisals (CPTCI)	37.9 (14.6)	.76****	197	.73****	196	.56****	197	.61****	197
Rumination	7.49 (2.8)	.62****	196	.58****	195	.41****	197	.42****	197
Self‐blame	3.5 (2.0)	.07	196	.02	195	−.07	196	−.02	196

CPSS, Child PTSD Symptom Scale; CPTCI, Child Post‐Traumatic Cognitions Inventory; MSPSS, Multidimensional Scale of Perceived Social Support; PTSS, post‐traumatic stress severity; SMFQ, Short Mood and Feelings Questionnaire; TMQQ, Trauma Memory Quality Questionnaire.

*p < .05; **p < .01; ***p < .001; ****p < .0001.

A second assessment was completed by 208 participants (92.0% of those who completed the 2‐ to 4‐week assessment) 2 months post‐trauma (*M* = 67.5 days, *SD* = 11.7). There were no differences between youth who did or did not complete the 2‐month assessment in terms of sex, age or initial traumatic stress symptoms (*p*s > .15).

### Measures

Demographic data or data pertaining to injury severity were gathered from the ED or initial interview.

#### Outcome measures

Diagnoses of *ASD and PTSD* were assessed using the Children's PTSD Inventory (CPTSDI). The CPTSDI is a youth‐report structured interview that possesses good internal consistency, inter‐rater reliability, test–retest reliability, convergent validity and discriminant validity (Saigh et al., 2000; Yasik et al., 2001). Additional items (available from the first author) were used to assess for dissociation symptoms (Meiser‐Stedman, Yule, Smith, Glucksman, & Dalgleish, 2005) and new symptoms relating to ‘negative alterations in cognition and mood’ in the DSM‐5. Thus, both DSM‐IV and DSM‐5 algorithms for ASD and PTSD were evaluated. *PTSS* was assessed using the Children's PTSD Symptom Scale (CPSS)(Foa, Johnson, Feeny, & Treadwell, 2001), a 17‐item self‐report measure. *Depression severity* was assessed using the 13‐item Short Mood and Feelings Questionnaire (SMFQ)(Wood, Kroll, Moore, & Harrington, 1995). For some analyses, a validated cut‐off of 16 was used on the CPSS to denote clinically significant PTSS (Nixon et al., 2013). All these measures have been validated for use with children and adolescents and were administered at the 2‐ to 4‐week and 2‐month assessments.

#### Predictor measures

Measures for assessing pre‐, peri‐ or post‐traumatic coping or functioning were administered at the 2‐ to 4‐week assessment. As far as possible, previously published measures were utilised that had been validated for use with children and adolescents. In addition to having good internal consistency, we selected measures that had evidence of construct validity; that is, they have been shown to be correlated with PTSS previously or have in some other way shown to index the construct they purport to represent. Where a suitable measure for a mechanism of interest was unavailable, the authors aimed to develop age‐appropriate measures. All such new measures are presented in Table S1 in the online Supporting Information section.

*Pretrauma emotional well‐being* was assessed with five items derived from the Post‐traumatic Adjustment Scale (O'Donnell et al., 2008) that indexed anxiety, low mood and anger.

Several aspects of participants’ peritraumatic cognitive state were assessed. *Perceived threat* was assessed using a three‐item scale devised in a previous study (Meiser‐Stedman, Dalgleish, Smith, Yule, & Glucksman, 2007). *Data‐driven processing* was assessed using the seven‐item Child Data‐Driven Processing Questionnaire (McKinnon et al., 2008). *Peritraumatic panic* was assessed using a questionnaire addressing each of the 10 symptoms associated with a panic disorder diagnosis; symptoms were simply scored as being present/absent during the trauma. *Peritraumatic dissociation* (a four‐item measure) and pain (a single‐item measure) were evaluated using self‐report questionnaires devised for this study (see Table S1).

Post‐trauma psychosocial factors and cognitive processing were assessed via several self‐report measures. *Adaptive processing*, that is deliberate efforts to clarify mentally what occurred in the trauma on their own or with the support of friends or family, was assessed using a five‐item measure devised for this study. *Ongoing pain* was indexed using a single item. *Life stressors* a child may have experienced in the previous year (e.g. moving home) were assessed using a brief 15‐item interview administered to parents or caregivers. *Social support* was indexed using the 12‐item Multidimensional Scale of Perceived Social Support (Zimet, Dahlem, Zimet, & Farley, 1988). *Persistent dissociation* was assessed using a four‐item questionnaire. *Trauma memory characteristics* were assessed using the 11‐item Trauma Memory Quality Questionnaire (Meiser‐Stedman, Smith, Yule, & Dalgleish, 2007). *Negative trauma‐related appraisals* were assessed using the 25‐item Children's Post‐Traumatic Cognitions Inventory (CPTCI) (Meiser‐Stedman et al., 2009). The CPTCI comprises two subscales: ‘permanent and disturbing change’ and ‘fragile person in a scary world’. *Trauma‐related rumination* was assessed using three questionnaire items from a previous study (Meiser‐Stedman et al., 2014). Self‐blame was assessed using a novel two‐item scale.

The majority of these measures were re‐administered at 2 months, allowing for test–retest reliability statistics to be calculated. All scales for assessing pre‐, peri‐ or post‐traumatic coping or functioning had acceptable internal consistency (i.e. Cronbach's *α* > .72) in the present sample, with the exception of our peritraumatic dissociation measure which was only borderline acceptable (*α* = .65). Psychometric properties (internal consistency and test–retest reliability, where possible) for all measures are presented in Table S2. Means and standard deviations for all predictor measures are displayed in Table 1.

### Procedure

The study was approved by the UK National Research Ethics Service, Cambridgeshire 1 Research Ethics Committee (10/H0304/11). Informed assent/consent from the child and their parent/carer was required for participation. Assessments were conducted via the telephone by graduate‐level psychologists.

### Statistical analysis

Checks were made to ensure that the assumptions of regression models were met. Scatterplots suggested that the relationships between independent variables and dependent variables were linear in nature. Residuals were normally distributed, supporting the assumption of multivariate normality. There was some evidence of heteroscedasticity; therefore, nonparametric adjustments were made by using bootstrapping (Chernick, 2008). Bootstrapping approximates what estimates might be generated if the whole population was sampled by repeatedly resampling the study sample; 1,000 resamples were used. Collinearity statistics were inspected for each regression model; there was no evidence of significant multicollinearity (i.e. no tolerance statistics <.2, no Variance Inflation Factors > 5). For significant one‐way analysis of variance (ANOVA) models, Hochberg post hoc comparisons were undertaken. The Welch test was used for ANOVA when there was significant heterogeneity of variance, with the Games‐Howell post hoc comparison. A structural equation model was evaluated in R 3.4.2 using the Lavaan package (Rosseel, 2012). Good model fit was indicated by a Comparative Fit Index (CFI) > .95, a Tucker‐Lewis Index (TLI) > .95, a root mean square error of approximation (RMSEA) < .08 and a standardised root mean squared residual (SRMR) < .08.

## Results

### Course of post‐traumatic stress over the first 2 months post‐trauma

Thirty‐two youth (14.2%) met criteria for DSM‐5 ASD at Week 2; at 2 months, 20 participants (9.6%) met DSM‐5 criteria for PTSD. Individual symptom clusters were endorsed by much greater proportions of participants. Full data on diagnostic outcomes have been reported elsewhere (Meiser‐Stedman, McKinnon et al., 2017). The numbers of youth meeting diagnostic thresholds declined between 2–4 weeks and 2 months regardless of which diagnostic algorithm was used. At 2–4 weeks, 55 youth (26.4%; missing = 11) scored above the clinical cut‐off on the CPSS, while at 2 months 35 (16.8%; missing = 2) scored above cut‐off.

### Regression modelling of post‐traumatic stress

All predictor variable means, standard deviations and correlations with Week 2 to Week 4 and Month 2 CPSS scores, and Week 2 ASD and Month 2 PTSD caseness are presented in Table 1. Initial zero‐order correlations revealed that objective indices of trauma severity (number of injuries, sustaining a fracture, being seen in ‘resus’, sustaining an injury with permanent loss of function) were not significantly related to PTSS (on the CPSS) at 2 months; these variables were not investigated further. The only exception to this was that being assaulted (relative to other trauma types) was associated with greater CPSS scores. Demographic variables (age, gender, ethnicity, household income >£20,000) and some psychosocial variables (social support, self‐blame) were also not significantly related to PTSS and so were not investigated further. The same pattern of results was observed for correlates of PTSS at Week 2, with the exception that sustaining a head injury was also a significant correlate and having a household income greater than £20,000 was protective against higher CPSS scores. Head injury was therefore included in subsequent regression models, but given the disproportionately large amount of missing data associated with household income it was not included; exploratory analyses revealed that household income did not account for unique variance in any regression model.

Significant predictors of post‐traumatic stress were further examined using hierarchical linear regression modelling of CPSS scores at 2 months. In order to test the hypothesis that peri‐ and post‐trauma cognitive processing plays a significant role over and above the impact of other plausible psychosocial vulnerabilities in driving later symptoms, predictor variables were entered in the following steps: (a) pretrauma mental health difficulties; (b) trauma characteristics; (c) peritrauma cognitive processing; (d) post‐trauma psychosocial factors *not* specifically highlighted in cognitive models of PTSD; and (e) post‐trauma cognitive processing (see Table 2). All predictor variables were assessed at 2–4 weeks. While the first and second steps each significantly improved the model, cognitive variables (either during or post‐trauma) accounted for considerably more variance in PTSS on the CPSS at 2 months than the other steps, post‐trauma cognitive processes accounting for 21% of additional unique variance on the final step. On this final step, pretrauma emotional difficulties, being assaulted, sustaining a head injury, peritrauma panic, data‐driven processing, persistent dissociation and trauma‐related appraisals accounted for unique variance in PTSS. A similar approach to modelling PTSS at 2–4 weeks was undertaken. The resultant model was significant, accounting for 75% of variability in acute symptoms on the CPSS, with subjective threat, peritraumatic pain, persistent dissociation, memory quality and negative appraisals each accounting for unique variance (see Table S3).

**Table 2:** Hierarchical regression model predicting post‐traumatic stress severity on the CPSS at 2 months post‐trauma

Predictor variable (assessed at 2–4 weeks)	Model		Step		Step 5		
	*Adj. R* ^*2*^	*F* test	Δ*R* ^*2*^	*F* test	B	Bootstrapped 95% CI	*β*
Step 1: Pretrauma factors	.10	*F*_1,189_ = 22.74, *p* < .0001	.10	*F*_1,190_ = 22.74, *p* < .0001			
Emotional difficulties					**−0.50**	**−0.87, −0.11**	−**.16**
Step 2: Trauma characteristics	.17	*F*_3,187_ = 13.57, *p* < .0001	.07	*F*_2,187_ = 8.12, *p* < .0001			
Assault versus nonassault					**3.07**	**0.10, 5.90**	**.12**
Head injury					−**3.42**	−**5.39,** −**1.55**	−**.17**
Step 3: Peritrauma cognitive processing	.42	*F*_8,182_ = 18.12, *p* < .0001	.27	*F*_5,182_ = 17.31, *p* < .0001			
Subjective threat					−0.30	−0.74, 0.15	−.08
Panic					0.57	−0.02, 1.16	.14
Data‐driven processing					**0.20**	**0.00, 0.41**	**.13**
Peritraumatic dissociation					0.08	−0.32, 51	.03
Peritraumatic pain					−0.79	−1.61, 0.13	−.09
Step 4: Post‐trauma psychosocial factors	.43	*F*_11,179_ = 13.87, *p* < .0001	.02	*F*_3,183_ = 1.85, p = .14			
Adaptive processing					0.04	−.21, 0.29	.02
Ongoing pain					0.17	−1.08, 1.45	.02
Life stressors					−0.10	−0.99, 0.64	−.01
Step 5: Post‐trauma cognitive processing	.64	*F*_15,175_ = 23.59, *p* < .0001	.21	*F*_4,175_ = 27.63, *p* < .0001			
Ongoing dissociation					**0.87**	**.19, 1.57**	**.21**
Memory quality (TMQQ)					0.11	−.09, 0.29	.07
Trauma‐related appraisals (CPTCI)					**0.32**	**.19, 0.43**	**.48**
Trauma‐related rumination					**0.42**	**.13, 0.83**	**.12**

Regression coefficients (B and *β*) where the 95% bootstrapped regression coefficient did not cross zero are highlighted in bold. CPSS, Child PTSD Symptom Scale; CPTCI, Child Post‐Traumatic Cognitions Inventory; TMQQ, Trauma Memory Quality Questionnaire.

In order to assess the hypothesis that cognitive processes have a role in maintaining post‐traumatic stress symptoms once they have become established, this 2‐month model was repeated but with Week 2 acute PTSS (CPSS scores) forced into an initial step. Acute PTSS alone accounted for 51% of variance in 2‐month PTSS (*F*_1,189_ = 199.24, *p* < .0001); the final model accounted for 65% of variance (*F*_16,174_ = 23.26, *p* < .0001), with acute PTSS (*β* = .23), pretrauma emotional difficulties (*β* = −.16), sustaining a head injury (*β* = −.19), data‐driven processing (*β* = .12), peritraumatic pain (*β* = −.11) and trauma‐related appraisals (*β* = .40) accounting for unique variance (see Table S4). Variables found to significantly contribute to this model were also entered into a logistic regression model with PTSD caseness at 2 months as the dependent variable. Variables were entered in the steps outlined above. Only trauma‐related appraisals accounted for further variance over and above the effect of acute PTSS on the CPSS (block *χ* ^2^ = 12.86, *p* < .0001; see Table S5).

A further regression was undertaken to test the possibility that negative appraisals may reflect a (possibly pretrauma) cognitive bias associated with depressive symptoms, that is our measure of negative appraisals may act as a proxy measure of depression, which is actually the factor predicting subsequent PTSS. This model examined whether 2‐ to 4‐week negative appraisals accounted for unique variance in 2‐month CPSS scores over and above the effect of 2‐ to 4‐week PTSS (on the CPSS) and severity of depression symptoms on the SMFQ (which were entered in the first and second steps, respectively). Appraisals, entered in the third step, significantly improved the model and accounted for unique variance in the final step (see Table S6 for full model), while depression did not account for any unique variance. This suggests that the effect of early appraisals was not driven by this variable functioning as a proxy for depression.

### Differentiating onset and maintenance factors

In order to distinguish the onset and maintenance processes involved in the early course of post‐traumatic stress, participants were assigned to groups on the basis of their CPSS scores (i.e. our self‐report questionnaire measure of post‐traumatic stress severity) to denote ‘caseness’, using the cut‐off scores noted above. With this approach, there were 134 ‘resilient’ cases (below cut‐off at both assessments), 27 ‘recovered’ cases (i.e. above cut‐off at 2–4 weeks, but not later) and 28 ‘persistent’ cases (above cut‐off at each assessment). The use of structured interview diagnostic assessments of ASD and PTSD caseness to allocate participants to each trajectory were frustrated by relatively small numbers of participants in the recovery and persistent groups (*n* = 15 and *n* = 14, respectively), which would have made any comparisons unreliable. A small number of ‘late onset’ participants (*n* = 6) did not score above cut‐off initially but did so at 2 months; given how small this group was, it was not considered in the formal analysis.

Results for all demographic, trauma‐related, psychosocial and cognitive processes variables, differentiated by trajectory, are displayed in Table 3. Demographic variables were unrelated to trajectory. Most objective indices of trauma severity were either unrelated to trajectory or, in the case of admission, more strongly associated with the resilient trajectory. Chi‐square tests found that being assaulted was related to trajectory; inspection of residuals suggested that youth who had been assaulted were more likely than expected to be in the persistent trajectory group. Post hoc comparisons for pretrauma emotional difficulties found that the recovery and persistent groups scored more highly on this measure relative to the resilient trajectory youth. On all measures of peritrauma cognitive processing, the recovery and persistent trajectory groups scored more highly than the resilient trajectory; for panic and dissociation, the persistent group scored more highly than the recovery youth. The recovery and persistent groups scored more highly on our measure of adaptive processing than the resilient group, while the persistent group endorsed more ongoing pain relative to the resilient group. While a significant between‐group effect was found for life stressors, no post hoc tests were significant. The persistent group scored more highly than the recovery group, who in turn scored more highly than the resilient group on all post‐trauma cognitive processing measures except self‐blame. The persistent group also scored higher on 2‐ to 4‐week measures of post‐traumatic stress severity and depression than the recovery group, who in turn scored higher than the resilient group.

**Table 3:** All predictor variables differentiated by trajectory of post‐traumatic stress severity (i.e. cut‐off on CPSS)

	ANOVA	Resilient (*n* = 134)		Recovery (*n* = 27)		Persistent (*n* = 28)	
		*M*	*SD*	*M*	*SD*	*M*	*SD*
Demographic factors							
Age	*F*_2,51.88_ = 2.81	14.0	3.0	14.7	2.8	15.1	2.3
Female Sex, *n* (%)	*χ* ^2^(2) = 5.82	52	(38.8%)	17	(63.0%)	14	(50.0%)
Minority ethnicity, *n* (%)	*χ* ^2^(2) = 0.42	8	(6.0%)	1	(3.7%)	1	(3.6%)
Income >£20K, *n* (%)	*χ* ^2^(2) = 11.64	93^a^	(78.2%)	10^b^	(43.5%)	16	(72.7%)
Pretrauma factors							
Emotional difficulties	*F*_2,39.44_ = 12.88***	6.87^a^	2.06	8.48^b^	3.25	11.04^b^	4.70
Trauma characteristics							
Assault, *n* (%)	*χ* ^2^(2) = 15.75***	17^a^	(12.7%)	3	(11.1%)	12^b^	(42.9%)
Head injury, *n* (%)	*χ* ^2^(2) = 5.36	46	(34.6%)	14	(51.9%)	15	(53.6%)
Number of injuries	*F*_2,186_ = 0.26	1.68	0.90	1.78	0.80	1.79	0.88
Fracture, *n* (%)	*χ* ^2^(2) = 3.18	36	(26.9%)	3	(11.1%)	6	(21.4%)
Admission, *n* (%)	*χ* ^2^(2) = 6.98*	46^a^	(34.3%)	6	(22.2%)	3^b^	(10.7%)
Resus, n (%)	*χ* ^2^(2) = 1.83	19	(14.4%)	2	(7.4%)	2	(7.1%)
Perm. loss function, *n* (%)	*χ* ^2^(2) = 2.98	7	(5.2%)	0	(0.0%)	0	(0.0%)
Peritrauma cognitive processing							
Threat	*F*_2,185_ = 21.49****	7.08^a^	2.47	9.44^b^	2.06	9.61^b^	1.85
Data‐driven processing	*F*_2,185_ = 32.44****	13.83^a^	5.29	18.85^b^	5.49	21.82^b^	4.73
Panic	*F*_2,185_ = 48.39****	2.82^a^	2.00	4.33^b^	1.82	6.68^c^	1.70
Peritraumatic dissociation	*F*_2,185_ = 34.54****	3.06^a^	2.65	5.52^b^	2.47	7.29^c^	2.73
Peritraumatic pain	*F*_2,60.79_ = 15.93****	2.81^a^	1.15	3.67^b^	0.62	3.43^b^	0.88
Post‐trauma psychosocial factors							
Adaptive processing	*F*_2,185_ = 6.02**	12.95^a^	3.78	15.07^b^	3.46	14.89^b^	3.13
Ongoing pain	*F*_2,44.72_ = 6.87**	1.50^a^	0.76	1.89	0.80	2.18^b^	1.06
Life stressors	*F*_2,185_ = 3.06*	0.74	1.05	1.07	1.27	1.25	1.21
Social support (MSPSS)	*F*_2,186_ = 0.38	69.92	13.33	68.00	13.17	68.21	11.41
Post‐trauma cognitive processing							
Persistent dissociation	*F*_2,37.13_ = 27.79****	0.58^a^	1.04	2.33^b^	2.69	4.79^c^	3.22
Memory quality (TMQQ)	*F*_2,185_ = 81.13****	18.71^a^	4.84	26.67^b^	5.87	30.82^c^	5.41
Negative appraisals(CPTCI)	*F*_2,38.42_ = 63.54****	31.19^a^	5.95	43.22^b^	10.54	63.64^c^	16.91
Rumination	*F*_2,83.62_ = 57.26****	6.47^a^	2.45	8.85^b^	2.09	10.93^c^	1.44
Self‐blame	*F*_2,185_ = 0.34	3.47	2.07	3.78	2.01	3.68	1.87
Mental health at 2–4 weeks							
CPSS (w2)	*F*_2,40.30_ = 189.61****	5.33^a^	4.36	23.37^b^	6.28	29.93^c^	8.81
SMFQ (w2)	*F*_2,39.42_ = 53.63****	2.78^a^	3.09	9.04^b^	5.06	13.93^c^	6.61

Superscript letters represent significant between‐group differences. CPSS, Child PTSD Symptom Scale; CPTCI, Child Post‐Traumatic Cognitions Inventory; MSPSS, Multidimensional Scale of Perceived Social Support; SMFQ, Short Mood and Feelings Questionnaire; TMQQ, Trauma Memory Quality Questionnaire.

*p < .05, **p < .01, ***p < .001, ****p < .0001.

There were concerns that the relatively small size of the recovery and persistent groups meant that some of our findings here were potentially underpowered. A post hoc power evaluation suggested that two‐tailed between‐group tests between the recovery and persistent groups were only adequately powered to detect a large (Cohen's *d* = .8) effect size (achieved power 83%, alpha = .05). We therefore calculated the effect sizes for all the comparisons made above for continuous variables (see Table S7). When considering the recovery versus persistent comparisons, only two variables yielded medium size differences that were not statistically significant (i.e. pretrauma emotional difficulties, peritraumatic data‐driven processing), suggesting that lack of power did not substantially skew our findings. Indeed, many of the key between groups differences were large (negative appraisals [*d* = 1.45], rumination [*d* = 1.16], persistent dissociation [*d* = .83]).

### Further test of the role of negative trauma‐related appraisals using structural equation modelling

A further test of the role of negative appraisals in persistent PTSS comprised a structural equation modelling (SEM) approach. In SEM, measurement error and covariances between disturbance terms can be accounted for, and more accurate path estimates produced. This procedure also partially replicated the modelling approach used by Palosaari et al. (2013). This model used a measurement model for PTSS and appraisals at 2–4 weeks and 2 months, with the structural model including autoregressive and cross‐lagged paths. A four‐factor measurement model was used to derive a latent variable for PTSS, based on existing factor‐analytic models in youth. The optimal model in this case was one where 2‐ to 4‐week appraisals predicted both appraisals and PTSS at 2 months; 2‐ to 4‐week PTSS did not predict either outcome, and the cross‐lagged path from PTSS had to be excluded from the model to obtain adequate fit (CFI = .982, TLI = .972, RMSEA = .065, SRMR = .033; see Figure S1).

## Discussion

This prospective longitudinal study of recently trauma‐exposed 8‐ to 17‐year‐olds considered the role of cognitive psychological processes in the onset and maintenance of PTSS in this age group in the context of other trauma‐related, demographic and psychosocial predictors. To our knowledge, no previous study has considered the range of both peritraumatic and post‐traumatic cognitive processes alongside other credible predictor variables, or focused on the crucial post‐trauma period (i.e. when a likely chronic pattern of nonrecovery may first be distinguished from youth whose acute stress symptoms recover without intervention). Some trauma‐related and demographic risk factors for PTSS were apparent, particularly pretrauma self‐reported emotional difficulties and being exposed to an assault. However, cognitive processes during the trauma and afterwards were the most powerful predictors of the onset and maintenance of PTSS, across cross‐sectional, regression and trajectory analyses. Moreover, core processes such as negative appraisals were found to account for variability in 2‐month PTSS over and above baseline PTSS and even baseline depression symptoms.

Some evidence of specificity was evident when considering the role of cognitive psychological processes in the three trajectories considered here. The *onset* of acute clinically significant traumatic stress symptoms was associated with perceived threat and data‐driven processing, while the *maintenance* or persistence of PTSS at the 2‐month follow‐up was associated with more poorly elaborated, sensory‐based trauma memories, persistent dissociation, trauma‐related rumination and more negative appraisals of the trauma. Other credible psychosocial factors were not significantly related to later PTSS, including life stressors, social support and self‐blame. This suggests that the strong relationships for cognitive processes were not simply a function of a more general tendency to endorse any items measuring maladaptive processes on our set of measures, and underlines the importance of identifying disorder‐specific risk factors. Deliberate attempts to process the trauma were associated with greater PTSS, rather than conferring any protection. An additional regression model suggested that the strong role for appraisals was not the result of an association with depressed mood. Moreover, when a structural equation model (that accounts for measurement error and yields more accurate path estimates) was used to model the relationship between acute appraisals and later PTSD, this path remained significant, consistent with the prospective longitudinal study of war‐affected children conducted by Palosaari et al. (2013).

### Theoretical implications

These findings are consistent with cognitive accounts of adult PTSD (Brewin et al., 1996; Dalgleish, 2004; Ehlers & Clark, 2000) and clarify how the course of early traumatic stress reactions in youth may be shaped by different cognitive psychological processes. Very early reactions to trauma in youth may be conceived of as a common reaction to the highly affect‐laden and hard‐to‐comprehend nature of trauma. In many cases, such reactions will diminish over the coming weeks, presumably through a number of mechanisms, for example desensitisation, elaboration of their trauma account. However, the presence of negative appraisals of the trauma and its sequelae (such as those captured by the CPTCI) disrupts these recovery processes.

Other theoretical issues are underscored here. While PTSD is characterised by high levels of cognitive and behavioural avoidance, in this youth cohort PTSD was paradoxically also associated with active deliberative thinking about the index trauma and its meaning, as indexed both by our measure of rumination and of what was hypothesised in advance to be ‘adaptive processing’. Children and adolescents may, like adults (Murray, Ehlers, & Mayou, 2002), dwell on questions around the meaning or causes of trauma, while striving to avoid certain specific reminders. The ‘recovery’ group of participants may have derived some benefit from the use of such strategies, but they do not appear to have helped the ‘persistent’ group. It is unclear whether such strategies are simply markers of distress or are actively counterproductive.

### Clinical implications

These data support targeting negative appraisals in the psychological prevention and treatment of PTSD. When viewed alongside data from randomised controlled trials that have found a role for such appraisals in mediating treatment response (Jensen, Holt, Mørup Ormhaug, Fjermestad, & Wentzel‐Larsen, 2018; Meiser‐Stedman, Smith et al., 2017; Pfeiffer, Sachser, de Haan, Tutus, & Goldbeck, 2017), the case for making maladaptive appraisals a focus of psychological treatment is strong. A further implication is the need to recognise and address ruminative thinking styles in youth affected by trauma, rather than assuming that the sole cognitive style adopted by youth is avoidance.

The potency of negative appraisals, alongside the other mechanism identified here, may also inform the development of interventions and screening tools for trauma‐exposed youth. Early interventions for youth (e.g. debriefing) have mostly been ineffective (Kramer & Landolt, 2011), though there is some limited evidence for information provision in youth with high levels of PTSS (Kenardy, Cox, & Brown, 2015). Future early interventions may be enhanced by a focus on the types of appraisals implicated here, and normalising the peritraumatic and post‐traumatic processes that give rise to acute traumatic stress.

To our knowledge, this is the first study to consider proactive efforts to process trauma (as indexed by our ‘adaptive processing’ measure) and we found that such efforts are associated with greater PTSS. We are hesitant to make any strong recommendations given the novelty of our findings and urge other investigators to consider this mechanism in future research. One possible clinical implication may be to recognise that some youth may be working very hard to make sense of their trauma, but such efforts may be counterproductive or futile. They may need support when attempting to process their experiences (e.g. from a caregiver or clinician) or may need encouragement to regulate the time they allocate to such processing; this may limit any impact on mood and free up for time for resuming activities the young engaged in pretrauma.

### Limitations

The sample included in this study was predominantly white British and had been exposed to a relatively narrow (albeit commonly occurring) range of traumas, for example no youth had been sexually assaulted or had been involved in a large‐scale disaster. The study's generalisability to other populations is therefore unclear. The study also relied on participant self‐report measures. However, by their very nature, objective measurement of most of the constructs considered is very difficult in such a large sample of trauma youth (e.g. narrative analysis to assess memory quality) or inappropriate (e.g. appraisals are inherently subjective). Moreover, we had to develop several new measures which therefore had unestablished construct validity. While the present sample was large relative to earlier work in this area, its size was inadequate to detect small effects. In particular, more incisive path analytic techniques may require a larger sample.

From a statistical perspective, a further criticism of the present study would be that our analytical procedures have focused on the level of between‐group effects and we were not able to consider within‐group effects. Future research will need larger samples and multiple waves of assessment to distinguish between‐group and within‐group effects, for example using random‐intercept cross‐lagged panel models.

## Conclusion

Children and adolescents exposed to single‐event traumatic stressors may have difficulties in processing the emotions and information directly associated with the trauma as it occurs, but this alone may not result in persistent clinically significant PTSS. The presence of negative appraisals around the trauma and its sequelae plays a central role in the onset and persistence of PTSS. PTSS in youth may be viewed as stemming from difficulties processing a wide variety of trauma‐related information and meanings.

**Key points**

- Children and young people exposed to single incident trauma underwent significant natural recovery with respect to PTSD symptoms between 2–4 weeks and 2 months post‐trauma.
- Other than being assaulted, objective indices of trauma severity were not related to the onset or maintenance of PTSD symptoms. Most demographic factors were not associated with the onset and maintenance of PTSD symptoms; low income was a weak predictor of PTSD symptoms but did not contribute to any regression models.
- The cognitive processes peritraumatic threat, data‐driven processing and pain were implicated in the *onset* of PTSD symptoms. The *maintenance* of PTSD symptoms was associated with peritraumatic dissociation and panic, and post‐trauma dissociation, trauma memory quality, rumination and negative appraisals.
- Comparison of recovery and persistent post‐traumatic stress groups, as well as regression modelling, demonstrated a strong role for negative trauma‐related appraisals in the maintenance of PTSD symptoms.
- Nonspecific psychosocial risk factors, for example self‐blame and social support, were unrelated to PTSD symptoms.

## Supporting information


**Table S1.** Novel questionnaires devised for the present study.
**Table S2.** Psychometric properties of study measures.
**Table S3.** Hierarchical regression modelling of post‐traumatic stress severity on the CPSS at 2–4 weeks.
**Table S4.** Hierarchical regression modelling of post‐traumatic stress severity on the CPSS at 2 months, controlling for 2‐ to 4‐week CPSS scores.
**Table S5.** Logistic regression modelling of PTSD diagnosis at 2 months, controlling for 2‐ to 4‐week CPSS scores.
**Table S6.** Hierarchical regression modelling of post‐traumatic stress severity on the CPSS at 2 months, considering the effect of 2‐ to 4‐week PTSD, depression and appraisals.
**Table S7.** Between‐group effect sizes for PTSS trajectories for all predictor variables.
**Figure S1.** Structural equation model of post‐traumatic stress and appraisals at Week 2 and 2 months.
