# Supplementary material for: A core role for cognitive processes in the acute onset and maintenance of post‐traumatic stress in children and adolescents
Source: J Child Psychol Psychiatry. 2019 Mar 25;60(8):875–84. doi: 10.1111/jcpp.13054 (PMC6711766; doi:10.1111/jcpp.13054)

**Supporting information – A core role for cognitive processes in the acute onset and maintenance of post-traumatic stress in children and adolescents – by Meiser-Stedman, et al.**

**Table S1.** Novel questionnaires devised for the present study.

| Scale | Items | Responses (scoring) |
| --- | --- | --- |
| Pre-trauma emotional well-being | 1. Before the event I used to feel really sad sometimes.  2. Before the event I sometimes felt very nervous or scared about things.  3. Before the event I had big problems with feeling very sad or scared.  4. Before the event I often got angry at people.  5. Before the event I often got upset and scared. | Not at all like that, A little bit like that, Like that, Very much like that  (1, 2, 3, 4) |
| Peritraumatic panic | While the event was happening...  1. ... did you have pain in your chest?  2. ... did you feel very hot or cold?  3. ... did you feel like you were choking, like you couldn't breathe?  4. ... did you feel dizzy or faint?  5. ... did you feel like you couldn't control yourself anymore, or that you were going mad?  6. ... did you heart beat very fast, or go boom-boom-boom very loud?  7. ... did you feel sick, like you might throw up?  8. ... did you sweat a lot?  9. ... did you find it hard to breathe?  10. ... did you shake or tremble? | No, Yes  (0, 1) |
| Peritraumatic dissociation | 1. Being so shocked that I didn’t feel anything  2. Not really noticing what was going on around me (like being in a bubble)  3. Feeling like I was in a dream  4. Feeling like I was outside my body, or that my body didn’t belong to me | Not at all, A little like that, Like that, Very much like that  (0, 1, 2, 3) |
| Peri-traumatic pain | How much pain were you in at the time of the [event]? | Not at all, A little, Some, A lot  (0, 1, 2, 3) |
| Adaptive processing | 1. I try to understand what happened during the event.  2. I try to piece it together in my mind.  3. I explain the whole story to my friends and family.  4. I think it through so that what happened makes sense in my mind.  5. I get help from my family and friends to understand the [event]. | Disagree a lot, Disagree a bit, Agree a bit, Agree a lot  (1, 2, 3, 4) |
| Persistent pain | Are you in pain at the moment? | Not at all, A little, Some, A lot  (0, 1, 2, 3) |
| Persistent dissociation | 1. Being so shocked that I didn’t feel anything  2. Not really noticing what was going on around me (like being in a bubble)  3. Feeling like I was in a dream  4. Feeling like I was outside my body, or that my body didn’t belong to me | Not at all or only one time, Once a week or less/once in a while, 2 to 4 times a week/half the time, 5 or more times a week/almost always  (0, 1, 2, 3) |
| Self-blame | 1. I made the event happen.  2. It was my fault the event happened. | Disagree a lot, Disagree a bit, Agree a bit, Agree a, lot  (1, 2, 3, 4) |

**Table S2.** Psychometric properties of study measures.

| Measure | Possible range | Internal consistency, this sample^a^ | Test-retest reliability,  this sample^b^ |
| --- | --- | --- | --- |
|  |  |  |  |
| *Outcome measures* |  |  |  |
| Children’s PTSD Symptom Scale (CPSS) | 0-51 | .92 | r(196)=.72, p<.0001 |
| Short Mood and Feelings Questionnaire (SMFQ) | 0-26 | .92 | r(195)=.76, p<.0001 |
|  |  |  |  |
| *Published predictor measures* |  |  |  |
| Peri-traumatic threat | 4-16 | .76 | r(194)=.73, p<.0001 |
| Child Data-Driven Processing Questionnaire (CDDPQ) | 7-28 | .88 | r(194)=.67, p<.0001 |
| Multidimensional Scale of Perceived Social Support (MSPSS) | 12-84 | .93 | r(195)=.53, p<.0001 |
| Trauma Memory Quality Questionnaire (TMQQ) | 11-44 | .84 | r(193)=.75, p<.0001 |
| Child Post-Traumatic Cognitions Inventory (CPTCI) | 25-100 | .95 | r(195)=.79, p<.0001 |
| Rumination | 3-12 | .76 | r(194)=.75, p<.0001 |
|  |  |  |  |
| *Unpublished predictor measures* |  |  |  |
| Pre-trauma emotional well-being | 5-20 | .83 | - ^c^ |
| Peri-traumatic panic | 0-10 | .72 | - ^c^ |
| Peri-traumatic dissociation | 0-12 | .65 | r(194)=.70, p<.0001 |
| Adaptive processing | 5-20 | .73 | r(194)=.51, p<.0001 |
| Persistent dissociation | 0-12 | .78 | r(195)=.50, p<.0001 |
| Self-blame | 2-8 | .90 | r(194)=.65, p<.0001 |

^a^ Cronbach’s alpha. ^b^ These measures were administered at the initial two-to-four assessment, then re-administered at two months, allowing for calculation of these test-retest reliability statistics. ^c^ Not collected

**Table S3.** Hierarchical regression modelling of post-traumatic stress severity on the CPSS at two-to-four weeks

|  | Model | |  | Step | |  | Step 5 | | |
| --- | --- | --- | --- | --- | --- | --- | --- | --- | --- |
| Variable | *Adj. R^2^* | *F* test |  | Δ*R^2^* | *F* test |  | B | Bootstrapped 95% CI | β |
| *Step 1: Pre-trauma factors* | .18 | F_1,190_ = 43.73, p<.0001 |  | .18 | F_1,190_ = 43.73, p<.0001 |  |  |  |  |
| Emotional difficulties |  |  |  |  |  |  | -.06 | (-.42, .32) | -.02 |
| *Step 2: Trauma characteristics* | .24 | F_3,191_ = 20.89, p<.0001 |  | .06 | F_2,188_ = 7.89, p<.001 |  |  |  |  |
| Assault vs non-assault |  |  |  |  |  |  | 2.11 | (-.52, 4.79) | .07 |
| Head injury |  |  |  |  |  |  | 1.48 | (-.78, 3.53) | .06 |
| *Step 3: Peri-trauma cognitive processing* | .55 | F_8,191_ = 30.50, p<.0001 |  | .32 | F_5,183_ = 27.44, p<.0001 |  |  |  |  |
| Subjective threat |  |  |  |  |  |  | **.59** | **(.13, .99)** | **.13** |
| Panic |  |  |  |  |  |  | .13 | (-.37, .66) | .03 |
| Data-driven processing |  |  |  |  |  |  | .02 | (-.19, .22) | .01 |
| Peritraumatic dissociation |  |  |  |  |  |  | .14 | (-.29, .56) | .04 |
| Peritraumatic pain |  |  |  |  |  |  | .81 | (.04, 1.62) | .08 |
| *Step 4: Post-trauma psychosocial factors* | .58 | F_11,180_ = 25.40, p<.0001 |  | .04 | F_3,180_ = 5.64, p<.001 |  |  |  |  |
| Adaptive processing |  |  |  |  |  |  | .16 | (-.08, .43) | .06 |
| On-going pain |  |  |  |  |  |  | .94 | (-.13, 2.24) | .07 |
| Life stressors |  |  |  |  |  |  | .42 | (-.78, 1.62) | .04 |
| *Step 5: Post-trauma cognitive processing* | .75 | F_15,191_ = 38.60, p<.0001 |  | .16 | F_4,176_ = 29.95, p<.0001 |  |  |  |  |
| On-going dissociation |  |  |  |  |  |  | **1.11** | **(.64, 1.58)** | **.24** |
| Memory quality (TMQQ) |  |  |  |  |  |  | **.27** | **(.00, .49)** | **.17** |
| Trauma-related appraisals (CPTCI) |  |  |  |  |  |  | **.24** | **(.13, .35)** | **.31** |
| Trauma-related rumination |  |  |  |  |  |  | .18 | (-.24, .60) | .05 |

^*^ p < .05; ^**^ p < .01; ^***^ p < .001. CPSS = Child PTSD Symptom Scale; CPTCI = Child Post-Traumatic Cognitions Inventory; TMQQ = Trauma Memory Quality Questionnaire. Regression coefficients (B and β) where the 95% bootstrapped regression coefficient did not cross zero are highlighted in bold.

**Table S4.** Hierarchical regression modelling of post-traumatic stress severity on the CPSS at two months, controlling for two-to-four week CPSS scores.

|  | Model | |  | Step | |  | Step 5 | | |
| --- | --- | --- | --- | --- | --- | --- | --- | --- | --- |
| Variable | *Adj. R^2^* | *F* test |  | Δ*R^2^* | *F* test |  | B | Bootstrapped 95% CI | β |
| *Step 1: Week two post-traumatic stress* | .51 | F_1,189_ = 199.24, p<.0001 |  | .51 | F_1,189_ = 199.24, p<.0001 |  |  |  |  |
| CPSS scores (week two) |  |  |  |  |  |  | **.20** | **(.02, .39)** | .23 |
| *Step 2: Pre-trauma factors* | .51 | F_2,188_ = 99.26, p<.0001 |  | .00 | F_1,188_ = .16, p=.69 |  |  |  |  |
| Emotional difficulties |  |  |  |  |  |  | **-.49** | **(-.86, -.11)** | -.16 |
| *Step 3: Trauma characteristics* | .53 | F_4,186_ = 54.32, p<.0001 |  | .03 | F_2,186_ = 5.08, p<.008 |  |  |  |  |
| Assault vs non-assault |  |  |  |  |  |  | 2.64 | (-.15, 5.31) | .10 |
| Head injury |  |  |  |  |  |  | **-3.72** | **(-5.62, -1.73)** | -.19 |
| *Step 4: Peri-trauma cognitive processing* | .57 | F_9,181_ = 29.36, p<.0001 |  | .06 | F_5,181_ = 4.87, p<.0001 |  |  |  |  |
| Subjective threat |  |  |  |  |  |  | -.42 | (-.91, .05) | -.11 |
| Panic |  |  |  |  |  |  | .54 | (.00, 1.13) | .13 |
| Data-driven processing |  |  |  |  |  |  | **.20** | **(.01, .40)** | .12 |
| Peritraumatic dissociation |  |  |  |  |  |  | .06 | (-.33, .45) | .02 |
| Peritraumatic pain |  |  |  |  |  |  | **-.96** | **(-.1.79, -.04)** | -.11 |
| *Step 5: Post-trauma psychosocial factors* | .57 | F_12,178_ = 21.82, p<.0001 |  | .00 | F_3,178_ = .28, p=.84 |  |  |  |  |
| Adaptive processing |  |  |  |  |  |  | .01 | (-.25, .27) | .00 |
| On-going pain |  |  |  |  |  |  | -.02 | (-.1.29, 1.21) | .00 |
| Life stressors |  |  |  |  |  |  | -.18 | (-1.09, .56) | -.02 |
| *Step 6: Post-trauma cognitive processing* | .65 | F_16,174_ = 23.26, p<.0001 |  | .09 | F_4,174_ = 11.75, p<.0001 |  |  |  |  |
| On-going dissociation |  |  |  |  |  |  | .65 | (-.09, 1.36) | .16 |
| Memory quality (TMQQ) |  |  |  |  |  |  | .05 | (-.16, .25) | .04 |
| Trauma-related appraisals (CPTCI) |  |  |  |  |  |  | **.27** | **(.13, .40)** | .40 |
| Trauma-related rumination |  |  |  |  |  |  | .39 | (-.04, .79) | .11 |

^*^ p < .05; ^**^ p < .01; ^***^ p < .001. CPSS = Child PTSD Symptom Scale; CPTCI = Child Post-Traumatic Cognitions Inventory; TMQQ = Trauma Memory Quality Questionnaire. Regression coefficients (B and β) where the 95% bootstrapped regression coefficient did not cross zero are highlighted in bold.

**Table S5.** Logistic regression modelling of PTSD diagnosis at two months, controlling for two-to-four week CPSS scores.

|  | Model | |  | Block |  | Block 5 | |
| --- | --- | --- | --- | --- | --- | --- | --- |
| Variable | Nagelkerke R^2^ | χ^2^ |  | χ^2^ |  | Wald statistic (df=1) | Odds ratio |
| *Block 1: Week two post-traumatic stress* | .54 | χ^2^(1)=55.70, p<.0001 |  | χ^2^(1)=55.70, p<.0001 |  |  |  |
| CPSS scores (week two) |  |  |  |  |  | 6.25^*^ | 1.13 |
| *Block 2: Pre-trauma factors* | .54 | χ^2^(2)=55.77, p<.0001 |  | χ^2^(1)=.07, p=.79 |  |  |  |
| Emotional difficulties |  |  |  |  |  | 4.95^*^ | .72 |
| *Block 3: Trauma characteristics* | .55 | χ^2^(3)=56.10, p<.0001 |  | χ^2^(1)=.33, p=.57 |  |  |  |
| Head injury |  |  |  |  |  | .02 | 1.10 |
| *Block 4: Peri-trauma cognitive processing* | .56 | χ^2^(5)=58.33, p<.0001 |  | χ^2^(2)=2.23, p=.33 |  |  |  |
| Data-driven processing |  |  |  |  |  | 1.25 | 1.10 |
| Peritraumatic pain |  |  |  |  |  | 2.24 | .50 |
| *Block 5: Post-trauma cognitive processing* | .67 | χ^2^(6)=71.18, p<.0001 |  | χ^2^(1)=12.86, p<.0001 |  |  |  |
| Trauma-related appraisals (CPTCI) |  |  |  |  |  | 12.41^****^ | 1.12 |

^*^ p < .05; ^**^ p < .01; ^***^ p < .001. CPSS = Child PTSD Symptom Scale; CPTCI = Child Post-Traumatic Cognitions Inventory; PTSD = post-traumatic stress disorder.

**Table S6.** Hierarchical regression modelling of post-traumatic stress severity on the CPSS at two months, considering the effect of two-to-four week PTSD, depression and appraisals.

|  | Model | |  | Step | |  | Step 3 | | |
| --- | --- | --- | --- | --- | --- | --- | --- | --- | --- |
| Variable | *Adj. R^2^* | *F* test |  | Δ*R^2^* | *F* test |  | B | Bootstrapped 95% CI | β |
| *Step 1: Week two post-traumatic stress* | .51 | F_1,194_ = 205.43, p<.0001 |  | .51 | F_1,194_ = 205.43, p<.0001 |  |  |  |  |
| CPSS scores |  |  |  |  |  |  | **.37** | **(.24, .51)** | .43 |
| *Step 2: Week two depression* | .52 | F_2,193_ = 108.01, p<.0001 |  | .01 | F_1,193_ = 5.66, p<.02 |  |  |  |  |
| SMFQ scores |  |  |  |  |  |  | -.19 | (-.50, .11) | -.12 |
| *Step 3: Week two appraisals* | .59 | F_3,192_ = 94.16, p<.0001 |  | .07 | F_1,192_ = 31.88, p<.0001 |  |  |  |  |
| CPTCI scores |  |  |  |  |  |  | **.33** | **(.22, .45)** | .50 |

^*^ p < .05; ^**^ p < .01; ^***^ p < .001. CPSS = Child PTSD Symptom Scale; CPTCI = Child Post-Traumatic Cognitions Inventory; SMFQ = Short Mood and Feelings Questionnaire. Regression coefficients (B and β) where the 95% bootstrapped regression coefficient did not cross zero are highlighted in bold.

**Table S7.** Between groups effect sizes for PTSS trajectories for all predictor variables.

|  | Effect size | | |
| --- | --- | --- | --- |
|  | Resilient vs Recovery | Resilient vs Persistent | Recovered vs Persistent |
| **Demographic factors** |  | | |
| Age | -0.24 | -0.41 | -0.16 |
| Female Sex^+^ | 2.68 | 1.58 | 1.70 |
| Minority ethnicity^+^ | 0.61 | 0.58 | 1.04 |
| Income >£20K^+^ | 0.26 | 0.59 | .44 |
| **Pre-trauma factors** |  |  |  |
| Emotional difficulties | -0.59 | -1.15 | -0.63 |
| **Trauma characteristics** |  |  |  |
| Assault^+^ | 0.86 | 5.16 | 0.17 |
| Head injury^+^ | 2.06 | 2.21 | 0.93 |
| Number of injuries | -0.12 | -0.12 | -0.01 |
| Fracture^+^ | 0.34 | 0.74 | 0.46 |
| Admission^+^ | 0.55 | 0.23 | 2.38 |
| Resus^+^ | 0.48 | 0.47 | 1.04 |
| Perm. loss function^+^ | 0.00 | 0.00 | - |
| **Peri-trauma cognitive processing** |  | | |
| Threat | -1.04 | -1.16 | -0.09 |
| Data-driven processing | -0.93 | -1.59 | -0.58 |
| Panic | -0.79 | -2.08 | -1.33 |
| Peri-traumatic dissociation | -0.96 | -1.57 | -0.68 |
| Peri-traumatic pain | -0.93 | -0.61 | 0.32 |
| **Post-trauma psychosocial factors** |  | | |
| Adaptive processing | -0.59 | -0.56 | 0.05 |
| On-going pain | -0.50 | -0.74 | -0.31 |
| Life stressors | -0.28 | -0.45 | -0.15 |
| Social support (MSPSS) | 0.14 | 0.14 | -0.02 |
| **Post-trauma cognitive processing** |  | | |
| Persistent dissociation | -0.86 | -1.76 | -0.83 |
| Memory quality (TMQQ) | -1.48 | -2.36 | -0.74 |
| Negative appraisals(CPTCI) | -1.41 | -2.56 | -1.45 |
| Rumination | -1.05 | -2.22 | -1.16 |
| Self-blame | -0.15 | -0.11 | 0.05 |
| **Mental health at 2-4 weeks** |  |  |  |
| CPSS (w2) | -3.34 | -3.54 | -0.86 |
| SMFQ (w2) | -1.49 | -2.16 | -0.83 |

^+^ = Odds ratios. All other effect sizes are Cohen’s d.

**Figure S1.** Structural equation model of post-traumatic stress and appraisals at week two and two months.

Note. Reexp = re-experiencing symptoms; Avoid = avoidance symptoms; Dysph = Dysphoric arousal symptoms; Hyper = Hyperarousal symptoms. Path coefficients (standard error)/standardised coefficient are presented for each pathway in the model, with p values for autoregressive and cross-lagged paths. Non-significant pathways are dashed. The 95% confidence intervals for the autoregressive and cross-lagged paths were: PTSD_wk2_-PTSD_wk8_, -.11, .29; Appraisals_wk2_-PTSD_wk8_, .20, .42; and Appraisals_wk2_-Appraisals_wk8_, .79, 1.01.


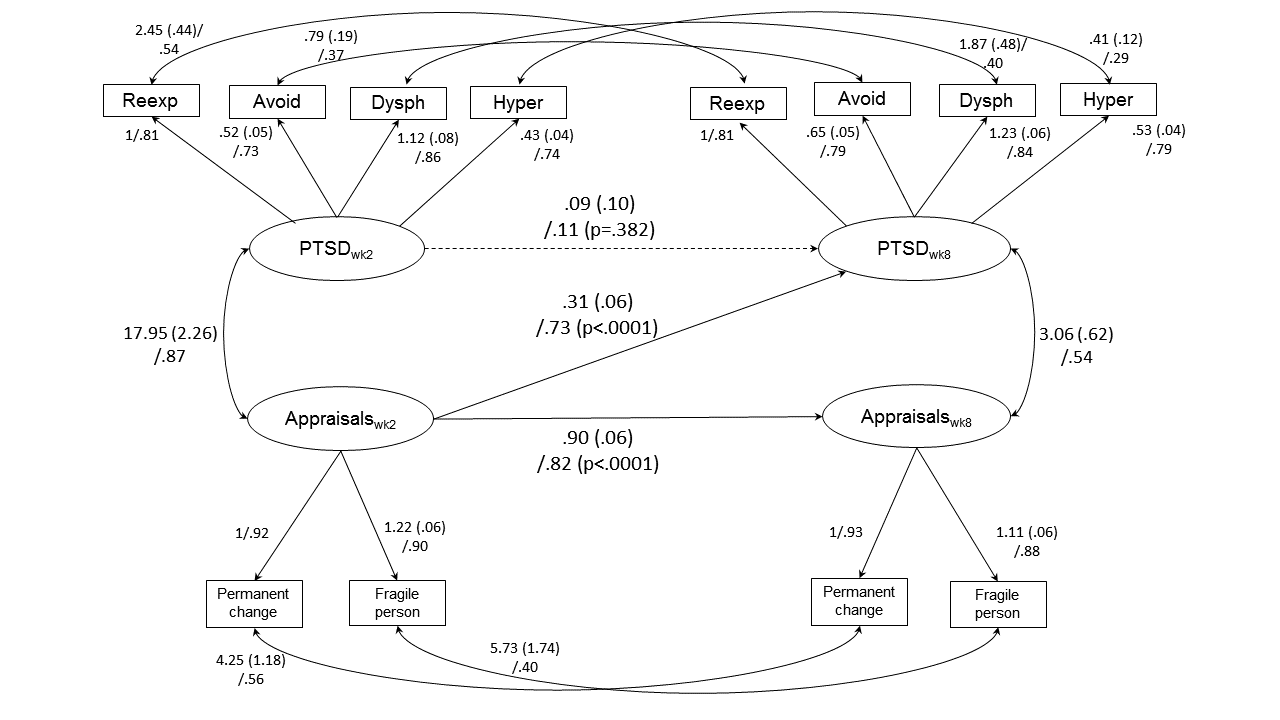

Supplement: Supplementary file 1 — Table S1. Novel questionnaires devised for the present study. Table S2. Psychometric properties of study measures. Table S3. Hierarchical regression modelling of post‐traumatic stress severity on the CPSS at 2–4 weeks. Table S4. Hierarchical regression modelling of post‐traumatic stress severity on the CPSS at 2 months, controlling for 2‐ to 4‐week CPSS scores. Table S5. Logistic regression modelling of PTSD diagnosis at 2 months, controlling for 2‐ to 4‐week CPSS scores. Table S6. Hierarchical regression modelling of post‐traumatic stress severity on the CPSS at 2 months, considering the effect of 2‐ to 4‐week PTSD, depression and appraisals. Table S7. Between‐group effect sizes for PTSS trajectories for all predictor variables. Figure S1. Structural equation model of post‐traumatic stress and appraisals at Week 2 and 2 months. [file JCPP-60-875-s001.docx]
